# Diffusion basis spectrum imaging as an adjunct to conventional MRI leads to earlier diagnosis of high-grade glioma tumor progression versus treatment effect

**DOI:** 10.1093/noajnl/vdad050

**Published:** 2023-04-19

**Authors:** Rowland H Han, Tanner M Johanns, Kaleigh F Roberts, Yu Tao, Jingqin Luo, Zezhong Ye, Peng Sun, Jacob Blum, Tsen-Hsuan Lin, Sheng-Kwei Song, Albert H Kim

**Affiliations:** Department of Neurological Surgery, Washington University School of Medicine, St. Louis, Missouri, USA; Division of Oncology, Department of Medicine, Washington University School of Medicine, St. Louis, Missouri, USA; The Brain Tumor Center, Siteman Cancer Center, Washington University School of Medicine, St. Louis, Missouri, USA; Department of Pathology and Immunology, Washington University School of Medicine, St. Louis, Missouri, USA; Division of Public Health Sciences, Department of Surgery, Washington University School of Medicine, St. Louis, Missouri, USA; Division of Public Health Sciences, Department of Surgery, Washington University School of Medicine, St. Louis, Missouri, USA; Department of Radiology, Washington University School of Medicine, St. Louis, Missouri, USA; Department of Radiology, Washington University School of Medicine, St. Louis, Missouri, USA; Department of Radiology, Washington University School of Medicine, St. Louis, Missouri, USA; Department of Radiology, Washington University School of Medicine, St. Louis, Missouri, USA; Department of Radiology, Washington University School of Medicine, St. Louis, Missouri, USA; Department of Neurological Surgery, Washington University School of Medicine, St. Louis, Missouri, USA; The Brain Tumor Center, Siteman Cancer Center, Washington University School of Medicine, St. Louis, Missouri, USA

**Keywords:** diffusion basis spectrum imaging, diffusion MRI, glioblastoma, pseudoprogression, radiation necrosis

## Abstract

**Background:**

Following chemoradiotherapy for high-grade glioma (HGG), it is often challenging to distinguish treatment changes from true tumor progression using conventional MRI. The diffusion basis spectrum imaging (DBSI) hindered fraction is associated with tissue edema or necrosis, which are common treatment-related changes. We hypothesized that DBSI hindered fraction may augment conventional imaging for earlier diagnosis of progression versus treatment effect.

**Methods:**

Adult patients were prospectively recruited if they had a known histologic diagnosis of HGG and completed standard-of-care chemoradiotherapy. DBSI and conventional MRI data were acquired longitudinally beginning 4 weeks post-radiation. Conventional MRI and DBSI metrics were compared with respect to their ability to diagnose progression versus treatment effect.

**Results:**

Twelve HGG patients were enrolled between August 2019 and February 2020, and 9 were ultimately analyzed (5 progression, 4 treatment effect). Within new or enlarging contrast-enhancing regions, DBSI hindered fraction was significantly higher in the treatment effect group compared to progression group (*P* = .0004). Compared to serial conventional MRI alone, inclusion of DBSI would have led to earlier diagnosis of either progression or treatment effect in 6 (66.7%) patients by a median of 7.7 (interquartile range = 0–20.1) weeks.

**Conclusions:**

In the first longitudinal prospective study of DBSI in adult HGG patients, we found that in new or enlarging contrast-enhancing regions following therapy, DBSI hindered fraction is elevated in cases of treatment effect compared to those with progression. Hindered fraction map may be a valuable adjunct to conventional MRI to distinguish tumor progression from treatment effect.

Key Points

- This is the first in vivo longitudinal, prospective study of diffusion basis spectrum imaging (DBSI) in brain tumor patients.
- DBSI hindered fraction is elevated in treatment-related enhancing lesions.
- DBSI may augment conventional MRI to diagnose treatment changes nearly 2 months earlier.

**Importance of the Study**

Prognosis for glioblastoma patients remains dismal despite advances in treatment. Magnetic resonance imaging findings after chemoradiotherapy, including perfusion and conventional diffusion-weighted imaging, can be ambiguous regarding effects of treatment versus true tumor progression. Diffusion basis spectrum imaging (DBSI) has been applied to predict underlying histopathology using ex vivo adult and pediatric brain tumor specimens. We now present the first in vivo longitudinal DBSI study for brain tumor patients and show that following treatment, new or enlarging contrast-enhancing lesions that exhibit an elevated hindered fraction represent treatment effect as opposed to progression. Furthermore, we find that DBSI may augment conventional imaging to make this diagnosis nearly 2 months earlier. Earlier discrimination between treatment-related changes and tumor progression would avoid discontinuation of effective treatment strategies or enable more timely transition to second-line therapies as appropriate, ultimately improving prognosis for this devastating disease.

Prognosis for glioblastoma patients remains dismal despite recent advances in treatment.^1–4^ After standard-of-care concurrent chemoradiotherapy, distinguishing effects of treatment from true tumor progression can be challenging using conventional MRI alone.^5,6^ Clinical decisions in these situations are often based on the evolution of radiographic changes on subsequent MR images.^7–9^ However, this strategy can lead to continuation of ineffective therapies that may prolong unchecked tumor growth. Furthermore, noninvasive identification of treatment-related imaging changes is of critical importance to avoid the inherent risks associated with additional biopsy or surgery.^10–12^ Commonly used imaging modalities including perfusion-weighted imaging and conventional diffusion tensor imaging (DTI) lack specificity and can be difficult to interpret.^13^ More advanced methods such as amino acid positron emission tomography (PET) have shown promising results but are not yet widely available.^14^ DBSI, which can be readily incorporated into standard MRI acquisitions without need for new equipment or reagents, may improve diagnostic accuracy and help guide treatment decisions for this devastating disease.

Diffusion-weighted MRI signals are sensitive to tissue structural characteristics and have been widely used to image tissue pathologies.^15,16^ However, they are affected by the strength and direction of the diffusion-sensitizing gradient. Thus, it would require a well-designed data acquisition scheme and corresponding tensor modeling to extract the structural characteristics revealed by these signals. To better extract structural information from diffusion-weighted MRI signals, DBSI uses a data-driven multiple-tensor modeling approach to disentangle the specific histologic components and structural features present within individual imaging voxels.^15,17–20^ Specifically, DBSI employs a multi-shell diffusion-weighting MRI acquisition and models tissue characteristics as a linear combination of discrete multiple anisotropic diffusion tensors (“fiber fraction”) and a spectrum of isotropic diffusion tensors (representing multiple extra-fiber structural components such as “vasogenic edema,” and “infiltrating inflammatory cells”). In this study, the isotropic diffusion fractions were referred to as “restricted” (representing inflammatory and/or tumor cells), “hindered” (vasogenic edema and/or necrosis), and “free diffusion” (cerebrospinal fluid and/or necrosis). DBSI-derived metrics better characterize tissue injury in a variety of central nervous system disorders including multiple sclerosis,^21,22^ cervical spondylotic myelopathy,^18^ spinal cord injury,^23^ optic neuritis,^19,24^ epilepsy,^25^ neonatal post-hemorrhagic hydrocephalus,^26^ obesity,^27,28^ and human immunodeficiency virus.^29^

Recent work has combined DBSI-derived structural metrics with a support vector machine algorithm to accurately predict areas with high tumor cellularity, necrosis, and tumor-infiltrated white matter in ex vivo human glioblastoma specimens.^16,30,31^ However, this previous study was limited by its cross-sectional nature and reliance on ex vivo samples obtained during surgical resection. The goals of the present longitudinal, prospective in vivo study are (1) to determine whether DBSI can noninvasively distinguish treatment effect from tumor progression in high-grade glioma (HGG) patients and (2) to determine if diagnosis can be achieved earlier with DBSI than with conventional MRI. Because DBSI hindered fraction is associated with tissue edema or necrosis,^16^ which are common histopathologic findings in pseudoprogression and radiation necrosis,^32^ we hypothesized that the hindered fraction map may complement conventional imaging for earlier diagnosis.

## Materials and Methods

### Study Design

This study was approved by the Institutional Review Board of the Washington University School of Medicine and conducted in accordance with the Declaration of Helsinki. Written informed consents were obtained from all participants. The first goal was to determine whether DBSI can noninvasively distinguish treatment effect from tumor progression in previously treated HGG patients. The second goal was to determine if diagnosis can be achieved earlier and with better specificity with DBSI than with standard-of-care MRI. Adult patients were prospectively recruited at Barnes-Jewish Hospital and Washington University School of Medicine if they had a known histologic diagnosis of HGG (WHO grade 3 or 4) and had completed standard-of-care radiation therapy with concurrent chemotherapy. Exclusion criteria included age less than 18 years, pregnant, contraindications for MRI, presence of paramagnetic metal implants, or were not able or willing to provide informed consent (or consent of legally authorized representative). All MRI data were collected during the study period from August 2019 to November 2020.

### Data Collection

DBSI and conventional MRI data were acquired longitudinally beginning with the standard-of-care 4-week post-radiotherapy MRI and continued with standard-of-care MRI until there was radiographic evidence of progression as defined by standard response assessment in neuro-oncology (RANO) criteria or progression confirmed by biopsy.^7^ Following progression, additional DBSI and conventional MRI were performed when clinically relevant in the opinion of the treating physician. If available, clinical MRI acquired outside of the DBSI scans were obtained to provide additional temporal context and disease course information. Conventional MRI included T1-weighted (T1W) with and without gadolinium (Gd), T2-weighted (T2W), fluid attenuated inversion recovery (FLAIR), DTI, and perfusion imaging. Although unprocessed diffusion-weighted data were available, final DBSI maps were not used in the real-time clinical decision-making of the enrolled subjects.

Clinical information for all subjects included age at initial diagnosis, sex, date of initial surgery, type of surgery, histopathologic diagnosis (WHO grade, *IDH* status, *MGMT* promoter status), and date of first bevacizumab use. Histopathologic diagnoses were updated based on WHO 5th edition by a neuropathologist (K.F.R.) when applicable. For data analysis, determination of “ground truth” treatment effect or progression diagnosis was made retrospectively with combination of clinical history, treatment decisions, radiologist interpretation of all available conventional MRI, and repeat resection or biopsy if clinically indicated. Time to standard-of-care diagnosis was the earliest date based on these methods, and time to DBSI-assisted diagnosis was the earliest date that may have been achievable using hindered fraction map in addition to standard-of-care. DBSI-assisted diagnosis was performed in a blinded fashion by a single author (R.H.H.) based on qualitative comparison of the hindered fraction in regions of concern with the surrounding brain parenchyma and supported by subsequent quantitative analysis, although a cutoff value was not used for this determination.

### DBSI Parameters

A 3-T Siemens Prisma with a 32-channel head coil was used for all DBSI MR acquisitions. Axial diffusion-weighted images (DWI) covering the whole brain were acquired using a multi-b-value diffusion-weighting scheme (99 directions; maximum b-value, 1500 s/mm^2^) as previously detailed^33^ with the following parameters: TR, 10 000 ms; TE, 120 ms; field of view 256 × 256 mm^2^; slice thickness, 2 mm; in-plane resolution, 2 × 2 mm^2^; and total acquisition time, 15 min. The 99 diffusion-encoding directions were selected as prescribed in diffusion spectrum imaging (DSI) where the position vectors are the entire grid points (qx, qy, qz) over the 3D q-space under the relationship (qx^2^ + qy^2^ + qz^2^) ≤*r*^2^, where *r* = 3 for DBSI while *r* = 5 for DSI. Patient motional artifacts in this cohort of subjects were not apparent. Nevertheless, eddy current and motion artifacts of DWI were corrected before susceptibility induced off-resonance field was estimated and corrected.

### DBSI Processing

DBSI models brain tumor diffusion-weighted MRI signals as a linear combination of discrete multiple anisotropic diffusion tensors and a spectrum of isotropic diffusion tensors:


$$\begin{array}{ll} \frac{S_{k}}{S_{0}}= & \sum_{i=1}^{N_{Aniso}}f_{i}e^{-\left|\vec{b_{k}}\right|\lambda_{⊥i}}e^{-\left|\vec{b_{k}}\right|\left(\lambda_{∥i}-\lambda_{⊥i}\right)\cos^{2}\phi_{ik}}\\ & +\int_{a}^{b}f\left(D\right)e^{-\left|\vec{b_{k}}\right|D}dD\left(k=1,\;2,\;3,\ldots \right). \end{array}$$

Here, *b*_*k*_ is the *k*th diffusion-weighting gradient. *S*_*k*_*/S*_*0*_ is the acquired diffusion-weighted signal at direction of *b*_*k*_ normalized to nondiffusion-weighted signal. *N*_*Aniso*_ is number of anisotropic tensors to be determined. Φ_*ik*_ is the angle between diffusion gradient *b*_*k*_ and principal direction of the *i*th anisotropic tensor. |*b*_*k*_| is *b*-value of the *k*th diffusion gradient. λ_∥*i*_ and λ_⊥*i*_ are axial and radial diffusivity of the *i*th anisotropic tensor under the assumption of cylindrical symmetry; ƒ_*i*_ is signal intensity fraction of the *i*th anisotropic tensor. *a* and *b* are low and high diffusivity limits of isotropic diffusion spectrum. ƒ*(D)* is signal intensity fraction at isotropic diffusivity *D*. DBSI analyses were performed using an in-house MATLAB script (MathWorks, Natick, MA; version 8.5, release R2015a) as previously detailed,^15,16^ with isotropic diffusion profiles defined as restricted fraction (0 ≤ *D* ≤ 1.0 μm^2^/ms, representing tumor cells), hindered fraction (1.0 < *D* ≤ 1.5 μm^2^/ms, representing necrosis or edema), and nonrestricted fraction (*D* > 1.5 μm^2^/ms, hindered fraction with diffusivity >1.5 μm^2^/ms, and free diffusion). The signal intensity fraction of anisotropic diffusion tensors represents the so-called fiber fraction. Generation of DBSI metric maps was blinded to “ground truth” tumor progression or treatment effect diagnoses in all cases.

### Quantitative Comparison of MRI Sequences

**Figure 1.**
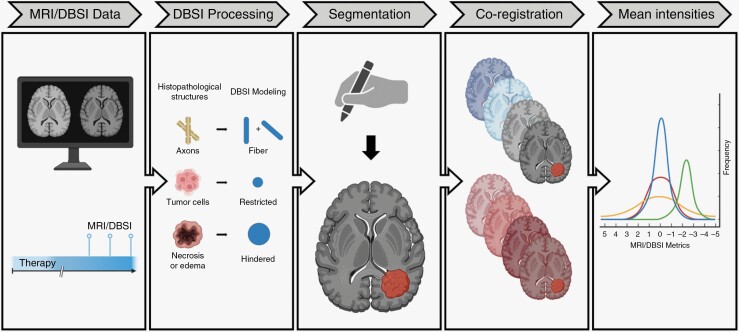
Illustrative quantitative analysis pipeline for conventional MRI and DBSI metrics. DBSI and conventional MRI data were acquired at multiple timepoints after radiotherapy. DBSI data were processed using a model that includes fiber, restricted, hindered, and nonrestricted fractions. At each timepoint, manual segmentation of new or enlarging contrast-enhancing lesions was performed on multiple axial slices. All available MRI sequences were co-registered to the T1-weighted post-gadolinium image (T1W-Gd) and tumor segmentation applied to each. Mean intensity over the segmented area for each MRI metric was recorded. Created with BioRender.com.

Conventional MR images and DBSI metric maps were co-registered to Gd-enhanced T1W (T1W-Gd) images at each individual timepoint using rigid transformation in ITK-SNAP version 3.8.0 (www.itksnap.org).^34^ For each T1W-Gd image, any new or enlarging contrast-enhancing lesions were manually segmented using ITK-SNAP on multiple axial slices. Voxels situated entirely within contrast-enhancing regions that were either smaller or absent on the most recent prior scan were selected, and this process repeated on every third axial slice until all lesions meeting the criteria were covered. The segmentations were applied to all co-registered images and mean intensities recorded. Intensity values at earliest DBSI-assisted diagnosis date based on qualitative review of images were compared between treatment effect and progression groups to determine any statistically significant differences (Figure 1).

### Statistical Analysis

Statistical analyses were performed using GraphPad Prism version 9.3.1 (San Diego, CA). Summary statistics are reported using median and interquartile range (IQR) or mean ± standard deviation (SD), as indicated. Two-tailed Welch’s unequal variances *t*-test was used for comparisons between 2 independent groups. Two-tailed Wilcoxon signed rank test was used for comparisons between matched samples. Categorical variables were compared using Fisher’s exact test. Statistically significant results are reported at a predetermined alpha level of 0.05, with Bonferroni correction for multiple comparisons when appropriate.

## Results

### Study Population

A total of 12 patients with HGG were enrolled in the study between August 2019 and February 2020, with final DBSI data obtained in November 2020. Demographic and histopathologic characteristics of the study population are detailed in Supplementary Table 1 and summarized here. Eight (66.7%) of the patients were male, with median age 56 years (range 40–71). Five (41.7%) of the patients had resection as their initial surgery, 6 (50%) had stereotactic biopsy, and 1 (8.3%) had biopsy with laser interstitial thermal therapy. Nine (75%) patients were diagnosed with WHO grade 4 glioblastoma, and 3 (25%) with WHO grade 3 or 4 glioma not meeting criteria for glioblastoma.

**Figure 2.**
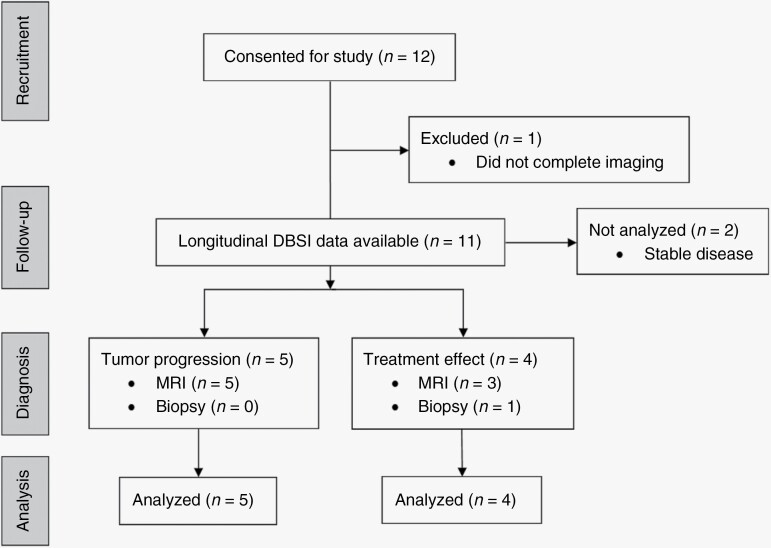
Consort diagram showing all recruited patients for the study and final diagnosis groups.

One patient was excluded because they withdrew from the study, and 2 patients without new or enlarging contrast-enhancing lesions during the study period were excluded from the present analysis, resulting in data from 9 patients included in the final study cohort. Figure 2 shows a consort diagram of the 12 enrolled patients and their outcomes. Of the 9 analyzed patients, 5 had tumor progression, and 4 had treatment effect based on standard practices. All but 1 case of treatment effect was diagnosed using standard interval MRI criteria, and the remaining patient (Subject C1-004) had 2 additional biopsies to confirm the diagnosis of treatment-related changes. All cases of tumor progression were determined using RANO radiographic criteria. Given the limited sample sizes, frequencies of biopsy versus resection (*P* = .5), glioblastoma versus nonglioblastoma histology (*P* = .4), WHO grade (*P* = .4), *MGMT* promoter status (*P* = 1.0), and patient sex (*P* = 1.0) were not significantly different between the tumor progression and treatment effect groups. All analyzed patients had *IDH*-wildtype tumors.

### DBSI Hindered fraction Distinguishes between Treatment Effect and Tumor Progression

**Figure 3.**
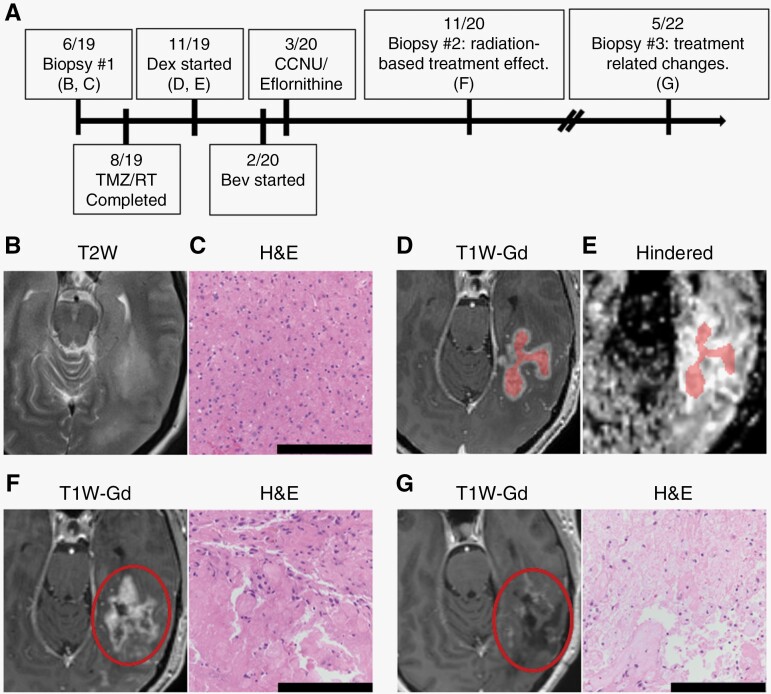
Imaging and histopathological findings for a case of treatment effect. (A) Treatment timeline for a 46-year-old female patient indicating timing of panels B to G. (B) Presenting MRI showed expansile T2 hyperintense lesions in the left cortex and subcortical white matter. (C) Initial biopsy H&E-stained section (20×) showing a diffusely infiltrative glial neoplasm without necrosis or microvascular proliferation. Two mitotic figures were identified in the limited biopsy specimen, leading to a diagnosis of diffuse glioma, high-grade, *IDH*-wildtype, NEC (updated based on WHO 5th edition; previously classified as WHO grade III anaplastic astrocytoma, *IDH*-wildtype). (D) MRI 3 months after radiation showed new multifocal areas of mass-like enhancement in the left temporal and occipital lobes at the site of previously treated tumor. (E) DBSI hindered fraction map on the same date as (D) showed homogeneous hyperintensity in the contrast-enhancing regions consistent with treatment effect. Segmented regions used in quantitative analyses are “highlighted”. (F) Repeat biopsy H&E-stained sections (20×) of an enlarging contrast-enhancing lesion demonstrated primarily bland necrosis with hyalinized blood vessels, features consistent with radiation necrosis. (G) Follow-up MRIs continued to show ambiguous contrast enhancement and a third biopsy 18 months after the second again showed treatment-related changes including bland necrosis, microvacuolation of neuropil, reactive gliosis, numerous macrophages, and hyalinized blood vessels with occasional scattered atypical cells. H&E, hematoxylin and eosin; NEC, not elsewhere classified; TMZ, temozolomide; RT, radiotherapy; Dex, dexamethasone; Bev, bevacizumab; CCNU, lomustine; T2W, T2-weighted; T1W-Gd, T1-weighted post-gadolinium. Scale bar = 200 µm.

**Figure 4.**
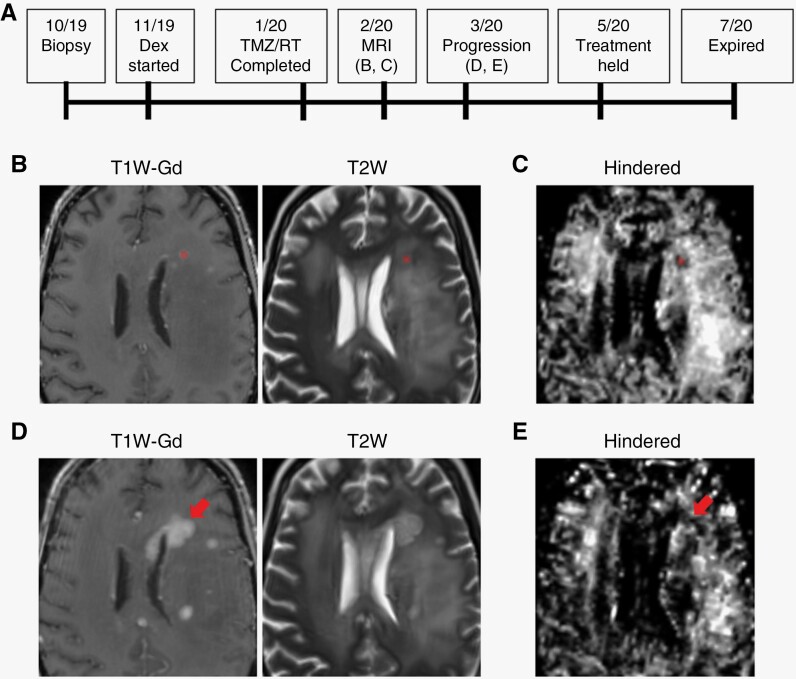
Serial imaging for a case of true tumor progression. (A) Treatment timeline for a 40-year-old female patient with diffuse astrocytic glioma with molecular features of glioblastoma, CNS WHO grade 4, indicating timing of panels B to E. (B) Post-radiotherapy MRI showed interval increased size of T2 signal abnormalities and new enhancing lesions. (C) DBSI hindered fraction map on the same date as (B) showed hypointensity in the regions of new contrast enhancement, which would have suggested disease progression. Segmented regions used in quantitative analyses are “highlighted”. (D) Two-month follow-up MRI confirmed tumor progression due to enlargement and interval development of new multifocal enhancing nodules with worsening T2 hyperintensity. (E) Follow-up DBSI hindered fraction map showed enlarging hypointensity in the same regions as (D) consistent with progression. Dex, dexamethasone; TMZ, temozolomide; RT, radiotherapy; T1W-Gd, T1-weighted post-gadolinium; T2W, T2-weighted.

Within new or enlarging contrast-enhancing regions, the DBSI hindered fraction map demonstrated hyperintensity (compared to surrounding brain parenchyma outside the regions of enhancement) for all cases of treatment effect and hypointensity for cases of tumor progression (Figures 3 and 4). In this limited dataset, there were no cases of discordance between DBSI hindered fraction findings and the final clinical or radiographic diagnosis. Although present, regional heterogeneity was less prominent in the hindered fraction maps compared to ADC and corresponded better to regions defined by new or enlarging contrast enhancement (data not shown).

**Figure 5.**
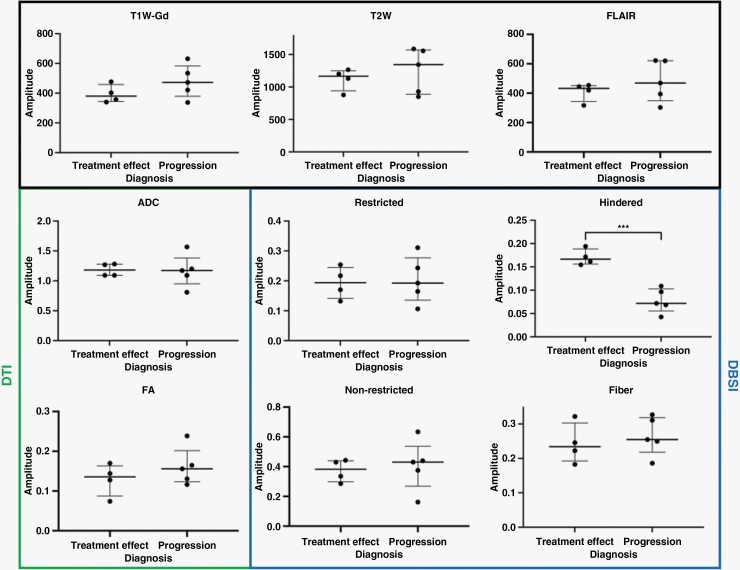
Comparison of conventional MRI and DBSI metrics between treatment effect (*n* = 4) and tumor progression (*n* = 5) groups. Lines are drawn at median and interquartile range. ***P < .001; 2-tailed unequal variances *t*-test. T1W-Gd, T1-weighted post-gadolinium; T2W, T2-weighted; FLAIR, fluid attenuated inversion recovery; ADC, apparent diffusion coefficient; FA, fractional anisotropy.

Contrast-enhancing lesions were manually segmented and mean signal intensities for all available MRI metrics were collected from co-registered images. As shown in Figure 5, DBSI hindered fraction values were higher in the treatment effect group (0.17 ± 0.02, mean ± SD) compared to progression (0.08 ± 0.03) (*P* = .0004, unequal variances *t*-test, significant at the Bonferroni-adjusted significance level of 0.05/9 = 0.0056). No statistically significant differences were seen between the 2 groups in T1W-Gd, T2W, FLAIR, DTI (ADC and FA), or other DBSI measures (restricted, nonrestricted, and fiber fractions), both with and without Bonferroni correction. Hindered fraction values were not significantly associated with biopsy versus resection (*P* = .2), *MGMT* promoter status (*P* = .8), or patient sex (*P* = .8).

### DBSI Hindered Fraction Augments Standard-of-care MRI for Earlier Diagnosis

For the 9 patients with treatment effect or tumor progression, Table 1 lists the time from index surgery to diagnosis by conventional MRI (including DTI and perfusion imaging) versus time to diagnosis by DBSI hindered fraction. DBSI was able to provide an earlier diagnosis of either progression or treatment effect in 6 (66.7%) patients. For the entire cohort, DBSI-assisted diagnosis occurred at a median 7.7 (IQR = 0–20.1) weeks before standard-of-care methods. For cases of tumor progression, DBSI led to earlier diagnosis by median 7.7 (IQR = 0–15.0) weeks.

**Table 1.** Time from initial surgery to treatment effect or tumor progression diagnosis by DBSI versus SOC.

Subject	Diagnosis	Surgery to DBSI diagnosis (weeks)	Surgery to SOC diagnosis (weeks)	Difference (weeks)
C1-001	Stable	N/A	N/A	N/A
C1-002	Excluded	N/A	N/A	N/A
C1-003	Treatment effect	11.3	11.3	0.0
C1-004	Treatment effect	23.0	75.0	52.0
C1-005	Treatment effect	12.7	20.4	7.7
C1-006	Progression	32.1	52.3	20.1
C1-007	Treatment effect	14.9	65.6	50.7
C1-008	Progression	16.6	31.6	15.0
C1-009	Progression	15.7	23.4	7.7
C1-010	Progression	19.0	19.0	0.0
C1-011	Progression	12.4	12.4	0.0
C1-012	Stable	N/A	N/A	N/A

SOC, standard-of-care; N/A, not applicable.

Because bevacizumab is known to affect diffusion imaging metrics,^35,36^ earliest use was compared to date of DBSI-assisted diagnosis. DBSI-assisted diagnosis was made before initiation of bevacizumab for all analyzed patients. For the 4 patients (3 treatment effect and 1 progression) who had DBSI data available both before and after bevacizumab use, no significant differences were observed in either DBSI hindered fraction (*P* = .9, Wilcoxon matched pairs signed rank test) or ADC (*P* = .4) after its initiation. However, this study was not adequately powered to detect whether bevacizumab impacts the use of DBSI.

### Illustrative Cases

#### Patient C1-004 (treatment effect)

Patient C1-004 (summarized in Figure 3A) is a 46-year-old female who initially presented with vision changes and had an MRI performed at another institution which showed expansile T2 hyperintense lesions in the cortex and subcortical white matter of the left hippocampus, temporal, and medial occipital lobes (Figure 3B). She underwent initial stereotactic biopsy and was diagnosed with diffuse glioma, high-grade, *IDH*-wildtype, not elsewhere classified based on the updated WHO 5th edition (Figure 3C, previously classified as WHO grade III anaplastic astrocytoma, *IDH*-wildtype). The patient was then treated with standard-of-care temozolomide and radiation therapy without complications. Three months after radiation, she developed difficulty reading. MRI at this time showed new multifocal areas of mass-like enhancement in the left temporal and occipital lobes at the site of previously treated tumor with significant increase in surrounding vasogenic edema and associated with elevated cerebral blood volume (Figure 3D). These findings were interpreted as representing pseudoprogression versus true progression. DBSI hindered fraction map at this time showed homogeneous hyperintensity in the contrast-enhancing regions consistent with treatment effect (Figure 3E). The patient was started empirically on dexamethasone with symptom resolution. Subsequent staging MRIs showed improvement in enhancement and mass effect without cerebral blood volume abnormality.

Seven months after completing radiation, staging MRI showed new contrast enhancement concerning for progression versus treatment effect. DBSI hindered map at this time continued to show hyperintensity in the new contrast-enhancing region consistent with treatment effect. However, based on conventional imaging, the patient was treated with second-line lomustine and eflornithine for tumor progression with no improvement. Due to persistent question of possible pseudoprogression, bevacizumab was also trialed after 1 month of second-line therapy without sustained radiographic response. Subsequent staging MRIs continued to show enlarging contrast-enhancing lesion, eventually leading to repeat stereotactic biopsy 8 months after appearance of the initial lesion. Histopathological analysis of tissue obtained from the contrast-enhancing region demonstrated radiation treatment effect without features of robust recurrence or higher-grade neoplastic element (Figure 3F). Notably, diagnosis based on this biopsy was nearly 12 months after the earliest DBSI-assisted diagnosis of the same region using hindered fraction map. Follow-up MRIs continued to show ambiguous results, and she eventually underwent a third biopsy 18 months after the second which again showed treatment-related changes without evidence of recurrent glioma (Figure 3G). DBSI hindered fraction maps in the region of contrast enhancement showed homogeneous hyperintensity at all available timepoints and may have avoided need for second-line therapy or additional surgical procedures after the initial biopsy.

#### Patient C1-009 (tumor progression)

Patient C1-009 (summarized in Figure 4A) is a 40-year-old female who presented with seizures and MRI showing multiple isolated non-enhancing areas of T2/FLAIR hyperintensity in the left frontal lobe, left basal ganglia and left mesial temporal lobe (images unavailable). She underwent biopsy of a left hippocampal lesion that was consistent with WHO grade 4 diffuse astrocytic glioma with molecular features of glioblastoma based on polysomy chromosome 7 and monosomy chromosome 10, *IDH*-wildtype, *MGMT* promoter unmethylated. She completed chemotherapy and radiation approximately 3 months later, and at that time had an MRI which showed interval increased size of T2/FLAIR signal abnormalities and new enhancing lesions (Figure 4B). These changes were favored to represent pseudoprogression though true progression could not be ruled out. DBSI hindered fraction map at this time showed hypointensity in the regions of new contrast enhancement, which suggested disease progression (Figure 4C).

Close follow-up was recommended based on the conventional imaging results, and 1 month later the patient had confirmed tumor progression due to enlargement and interval development of new multifocal enhancing nodules with worsening T2 hyperintensity (Figure 4D). DBSI hindered fraction again demonstrated increased hypointensity in the same regions (Figure 4E). She was transitioned to second-line therapy (bevacizumab and lomustine) at that time. Two months later, she elected to hold treatment due to functional decline and passed away 2 months later. DBSI-assisted diagnosis would have occurred 7.7 weeks before standard-of-care imaging, which may have resulted in earlier transition to secondary treatment.

## Discussion

Standard-of-care treatment for glioblastoma is surgical resection followed by concurrent temozolomide chemotherapy and radiotherapy, which can often result in new or worsening contrast-enhancing lesions on follow-up conventional MRI that may represent treatment effect or true tumor progression.^13^ Most lesions are a mixture of the 2 diagnoses with high levels of regional heterogeneity, but the clinically relevant distinction between primarily viable tumor progression versus nonviable treatment changes are the focus of the present study. The current strategy for evaluating these lesions is close radiographic monitoring, which may lead to continuation of ineffective therapies if they indeed represent progression. Conversely, definitive diagnosis may require additional invasive surgery such as biopsy which carries inherent risks. With the advent of DBSI and recent application to adult glioblastoma,^16^ we sought to determine whether the hindered isotropic diffusion fraction, representing edema resulting from treatment-induced blood-brain barrier permeability or necrosis, may augment conventional MRI to diagnose treatment effect or progression more effectively.

Using longitudinal, prospectively gathered data on 9 HGG patients, we demonstrated that in regions with new or enlarging contrast enhancement on post chemoradiotherapy MRI, mean DBSI hindered fraction is significantly higher in cases of treatment effect compared to true tumor progression (Figure 5, *P* = .0004). No differences were found between the 2 groups for all other available MRI measures including T1W-Gd, T2W, DTI, and other DBSI fractions. Although we did not observe any statistically significant changes in DBSI hindered fraction or ADC after initiation of bevacizumab in the 4 patients with available data, this is likely related to limited sample size. Additionally, we found that diagnosis using the hindered fraction map in addition to standard-of-care would have occurred 7.7 weeks (median) before conventional MRI alone (Table 1). Given the poor survival outcomes for HGG patients, DBSI may improve diagnostic capabilities in the post-treatment setting in a clinically significant way, although further validation with expanded datasets is needed.

Several other advanced imaging methods are currently in use or being developed to assist with post-treatment diagnosis in HGG. Perfusion-weighted imaging and conventional DTI are commonly used in clinical practice to help make the diagnosis, but their ability to clearly distinguish treatment-related changes from disease progression remain controversial.^13,37,38^ This difficulty is in part due to regional heterogeneity with areas of mixed high tumor cellularity, necrosis, edema, and microhemorrhage in the post-treatment setting. Furthermore, perfusion MRI acquisition and analysis methods vary widely between centers and confound interpretability of findings. In the present study, we found that despite using both DTI and perfusion imaging as part of standard-of-care, treating clinicians including radiologists were not able to reliably distinguish treatment effect from progression in a timely manner for several patients. DBSI hindered fraction is also impacted by regional heterogeneity but appears to predict overall viable tumor progression better than conventional imaging alone. Like DTI, DBSI maps generated from clinical imaging data at the millimeter scale are representative of ensemble averages of diffusion and histological structures with an effective resolution in the 10 µm range, which is the displacement of water molecules within the diffusion time.

Other more advanced imaging methods including amino acid PET,^14^ single proton emission computed tomography,^39,40^ and proton MR spectroscopy^41^ are useful adjuncts but not readily available at many centers. One major advantage of DBSI is that the analysis can be applied with minor modifications to the existing diffusion-weighted acquisition scheme, which would make broad application more feasible. A center wanting to integrate DBSI into their clinical imaging protocols would simply need to acquire multi-shell DWI such as with the 99-direction scheme used in this study (or another multi-direction, multi-b-value scheme, eg, diffusion scheme used in the ABCD project)^42^ and apply the processing steps outlined above. Either a 3-T or 1.5-T scanner can acquire these data, and conventional DTI analysis can still be performed for comparison. The DBSI acquisition time can also be shortened by employing advanced multi-band data acquisition.

Although previous work has applied DBSI in ex vivo adult and pediatric brain tumor specimens to classify underlying histopathology,^16,43^ this represents the first longitudinal in vivo study of DBSI metrics in brain tumor patients. Importantly, patients were imaged at multiple time-points beginning with the standard 4-week post-radiation therapy MRI. Thus, we were able to determine that treatment effect or tumor progression diagnosis could be made sooner than with conventional MRI when information from DBSI hindered fraction is considered. However, it should be noted that in all but 2 of the analyzed patients, the earliest DBSI-assisted diagnosis was made on the date of first DBSI data acquisition. Therefore, DBSI data acquired earlier during HGG treatment, such as immediately post-operatively or during concurrent chemoradiotherapy, may reveal additional clinically relevant findings. Finally, DBSI may eventually become a valuable adjunct in the preoperative planning phase of treatment if improved tractography or prediction of underlying histopathology is achievable over conventional DTI.

In summary, we report the first longitudinal prospective study of DBSI as an adjunct to conventional MRI including perfusion imaging and DTI in adult HGG patients. We found that in new or enlarging contrast-enhancing regions post-treatment, the DBSI hindered isotropic diffusion is elevated in cases of treatment effect when compared to true tumor progression. When combined with conventional imaging, hindered fraction map can help diagnose treatment effect or progression on average nearly 2 months before conventional MRI alone. Future work is needed to prospectively validate our approach in larger cohorts of HGG patients at the time of clinical decision-making, determine sensitivity and specificity of DBSI for both tumor progression and treatment effect, and assess the clinical utility of DBSI at earlier stages of HGG care.

## Supplementary Material

vdad050_suppl_Supplementary_Table_S1
