# Supplementary material for: Diffusion basis spectrum imaging as an adjunct to conventional MRI leads to earlier diagnosis of high-grade glioma tumor progression versus treatment effect
Source: Neurooncol Adv. 2023 Apr 19;5(1):vdad050. doi: 10.1093/noajnl/vdad050 (PMC10195207; doi:10.1093/noajnl/vdad050)
Supplement: vdad050_suppl_Supplementary_Table_S1 [file vdad050_suppl_supplementary_table_s1.docx]

| **Supplementary Table 1: Demographics and histopathologic findings for the study cohort.** | | | | | | | |
| --- | --- | --- | --- | --- | --- | --- | --- |
| **Subject** | **Sex** | **Age at diagnosis** | **Initial surgery type** | **Pathology** | **WHO grade** | ***IDH* status** | ***MGMT* promoter** |
| C1-001 | F | 57 | LITT/biopsy | Glioblastoma | 4 | WT | Methylated |
| C1-002 | M | 47 | Resection | Astrocytoma | 4 | Mutant | Methylated |
| C1-003 | M | 67 | Biopsy | Glioblastoma | 4 | WT | Unmethylated |
| C1-004 | F | 46 | Biopsy | Diffuse glioma, NEC | 3/4 | WT | Unmethylated |
| C1-005 | M | 54 | Biopsy | Glioblastoma | 4 | WT | Methylated |
| C1-006 | M | 66 | Resection | Glioblastoma | 4 | WT | Unmethylated |
| C1-007 | M | 55 | Resection | Glioblastoma | 4 | WT | Unmethylated |
| C1-008 | M | 69 | Resection | Glioblastoma | 4 | WT | Methylated |
| C1-009 | F | 40 | Biopsy | Glioblastoma | 4 | WT | Unmethylated |
| C1-010 | M | 63 | Biopsy | Glioblastoma | 4 | WT | Unmethylated |
| C1-011 | F | 71 | Resection | Glioblastoma | 4 | WT | Inconclusive |
| C1-012 | M | 43 | Biopsy | Anaplastic astrocytoma | 3 | Mutant | Not tested |
| F, female; M, male; LITT, laser interstitial thermal therapy; WHO, World Health Organization; WT, wild-type; NEC, not elsewhere classified. | | | | | | | |
